# Synthesis and Characterization of Selenium Nanoparticles-Lysozyme Nanohybrid System with Synergistic Antibacterial Properties

**DOI:** 10.1038/s41598-019-57333-7

**Published:** 2020-01-16

**Authors:** Mahsa Vahdati, Tahereh Tohidi Moghadam

**Affiliations:** 1 Department of Biology, Science and Research Branch, Islamic Azad University, Tehran, Iran; 2 Department of Nanobiotechnology, Faculty of Biological Sciences, Tarbiat Modares University, Tehran, Iran

**Keywords:** Nanoscale biophysics, Nanoparticles

## Abstract

In the light of promising potency of selenium nanoparticles in biomedical applications, this is the first study to report the synergistic antibacterial activity of these nanoparticles and lysozyme. The nanohybrid system was prepared with various concentrations of each component. Resistance of *Escherichia coli* and *Staphylococcus aureus* was compared in the presence of individual *Nano* and *Bio* counterparts as well as the nanohybrid system. Upon interaction of SeNPs with Lysozyme, the nanohybrid system efficiently enhanced the antibacterial activity compared to the protein. Therefore, SeNPs play an important role in inhibition of bacterial growth at very low concentrations of protein; whereas very high amount of the protein is required to inhibit bacterial growth individually. On the other hand, lysozyme has also played a vital role in antibacterial property of SeNPs, inducing 100% inhibition at very low concentration of each component. Hence, presence of *both* nano and *bio* counterparts induced vital interplay in the Nanohybrid system. The aged samples also presented good stability of SeNPs both as the intact and complex form. Results of this effort highlight design of nanohybrid systems with synergistic antibacterial properties to overcome the emerging antibiotic resistance as well as to define fruitful applications in biomedicine and food safety.

## Introduction

There has been increasing concern in the era of antibiotic resistance as bacteria rapidly continue to develop adaptive countermeasures against conventional antibiotics^1^. Bacteria are potentially life-threatening agents, capable of promoting infectious diseases. The history of bacteria acting as causative agents for infection goes back to the 14^th^ century. Salvarsan was the first antimicrobial agent introduced in 1910. Soon after that, other antimicrobial agents such as chloramphenicol, nalidixic acid, and macrolides were used worldwide. The 20^th^ century experienced temporary relief to infectious bacterial pathogens. Nevertheless, overexposure to antibiotics and evolution of effective countermeasures against antibacterial agents led to the emergence of antibiotic-resistant bacteria^2^. Since then, significant efforts were focused on overcoming the emergence of these resistant strains by development of new antibiotic drugs boasting chemical diversity and identification of antibiotic-producing bacteria as well as additional antibiotics from natural, previously unexplored sources. Nonetheless, these advancements could not compensate the rapidly increasing number of resistant bacterial strains. Nanotechnology is widely used in the generation of diverse products in the fields of biology and medicine. Using nanotechnology in biology has provided many opportunities in many areas, including tissue engineering, drug delivery, diagnosis, imaging and fight against bacterial infections^3^. With the need for new antimicrobial agents, nanoparticles have been proposed to treat infections as they use different mechanisms to kill bacteria than conventional antibiotics^4^, with relatively low toxicity in human cells. As a result, Nanomaterials can be considered as a promising alternative to antibiotics to control bacterial infections^5^.

Selenium, a nutrient element that has a massive function in biological systems, is one of the interesting compounds to integrate with antibacterial agents. Selenium is an essential trace element in the diet, required for maintenance of health and growth^6^. Given that the least toxic form of Se is elemental Se^7^, its nano form has attracted significant attention^8^. In recent years, several studies have pointed out the ability of selenium nanoparticles to exhibit anticancer^9^, antioxidant^10^, antibacterial and anti-biofilm^11^ properties. So far, remarkable antimicrobial activity of these nanoparticles have been evidenced against pathogenic bacteria, fungi and yeasts^12–14^.

Lysozyme is a biomolecule that has been widely distributed in humans, vertebrates, plants, bacteria and phages, which plays an important defensive role in the innate immune system. It is known as an antibacterial enzyme that accelerates hydrolyzes of β1,4 glucosidic linkages between N-acetylglucosamine (NAG) and N-acetylmuramic acid (NAM) in peptidoglycan of the cell wall, especially in gram-positive bacteria^15^. Furthermore, there are evidences that lysozyme could be an effective agent for killing human immunodeficiency virus (HIV)^16,17^. Chicken egg white lysozyme (HEWL), as a highly abundant small globular protein, has a vast industrial use as safe food preservative^18,19^. HEWL has also been widely considered as a model to study its interaction with various nanomaterials such as gold nanorods^20^, nano diamonds^21^, silver^22^, ZnO^23^ and polymeric nanoparticles^24^.

Considering the natural antimicrobial effects of lysozyme, as well as the promising antimicrobial potential of selenium nanoparticles, the present study has been carried out to integrate selenium nanoparticles with lysozyme, based on the hypothesis that presence of the *nano* and *bio* counterparts in form of a Nanohybrid system might induce promising synergistic effect. Herein, the antibacterial activity of pristine SeNPs and lysozyme, as well as their complex form, has been investigated on standard strains of *S. aureus* and *E. coli*. To the best of our knowledge, this is the first effort to study the possible synergistic antibacterial effect of selenium nanoparticles and lysozyme.

## Results and Discussion

### Characterization of Se nanoparticles

In this study, a facile wet chemical method was utilized for synthesis of monodisperse SeNPs by Ascorbic acid with biocompatibility and good reducing properties. Selenium oxyanion SeO_3_^2^) was formed in aqueous medium. When this solution reacted with ascorbic acid, selenium was reduced to elemental selenium (Se^0^).

**Figure 1:**
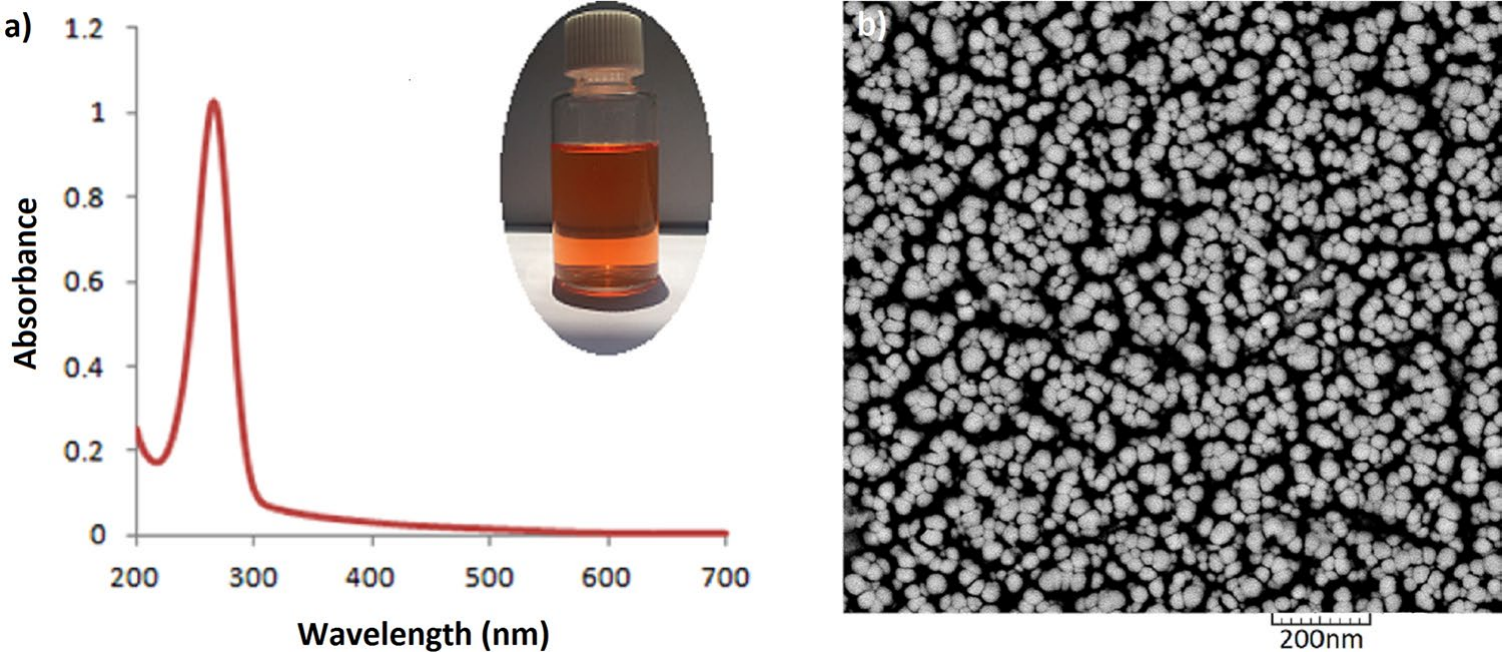
Absorption spectrum (**a**) and SEM image (**b**) of SeNPs. The inset shows color of the colloidal suspension.

The characteristic absorption peak of the Se nanoparticles is shown in Fig. 1a. Appearance of a sharp peak at 265 nm as well as the intense orange color of the colloidal dispersion (Fig. 1a, the inset) shows formation of Se nanoparticles. Characterization of the purified samples by scanning electron microscopy depicted the spherical morphology of the nanoparticles (Fig. 1b). Analysis of SEM by Image J software showed that size of the nanoparticles was 35.6 ± 7.5 nm.

### Characterization of SeNPs-Lysozyme (the Nanohybrid system)

**Figure 2:**
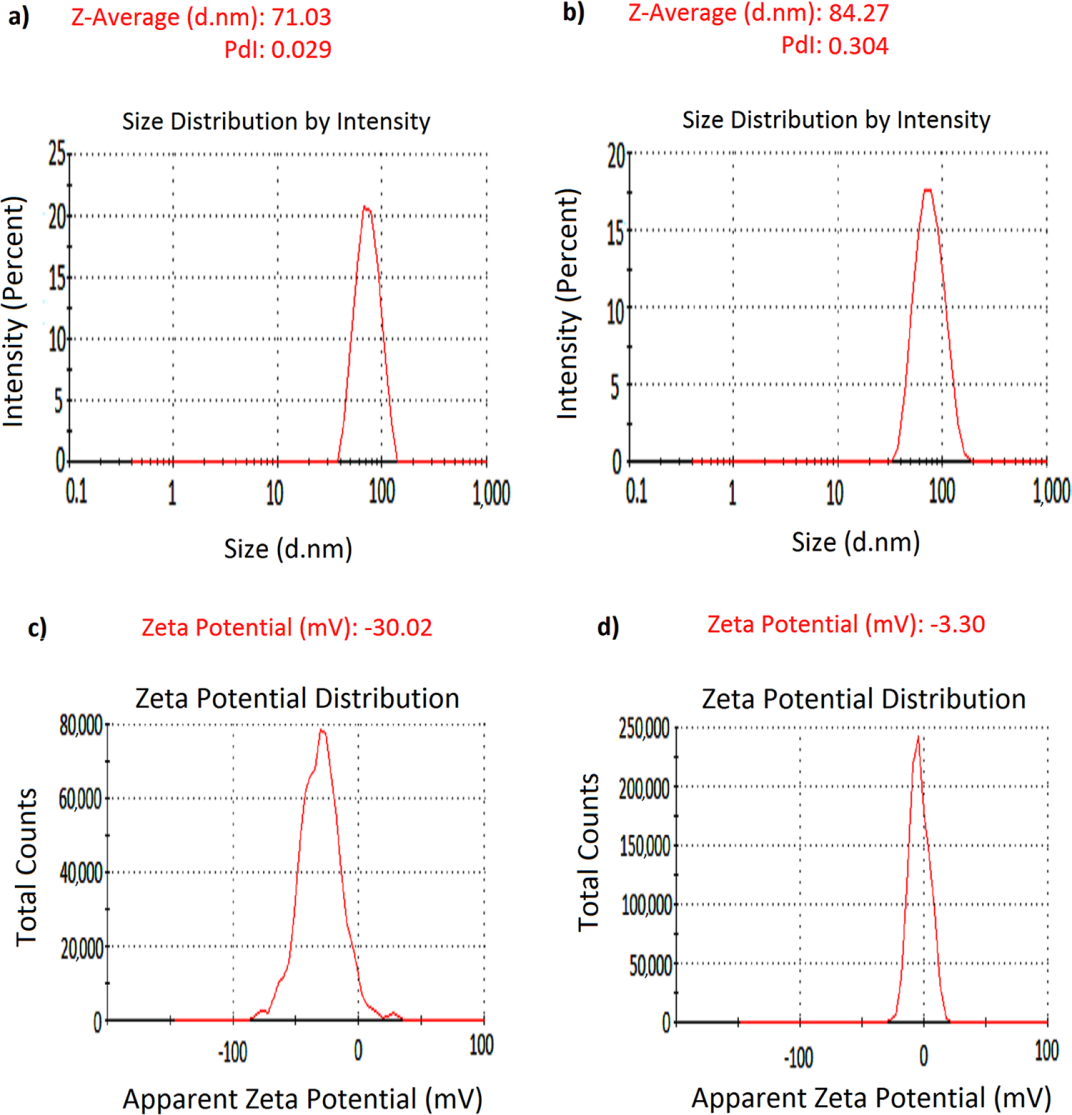
DLS measurements of SeNPs (**a**), and SeNPs-Lysozyme (100 µg/mL) (**b**), with Zeta potential of SeNPs (**c**), and Lysozyme-SeNPs (**d**), dispersed in 20 mM phosphate buffer, pH 6.2.

In order to monitor the interaction of nano and bio components, the dynamic light scattering (DLS) technique was used to compare hydrodynamic size of the purified SeNPs with their hybrid form. Figure 2 shows Z-average values of the SeNPs before and after interaction with lysozyme. Z-average size has become the accepted norm for presenting particle sizing results. Since the calculation of this parameter is mathematically stable, its result is insensitive to noise, making it a preferred DLS size parameter and a reliable measure of the average size of a particle size distribution. Z-average increases as the particle size increases. In the present study, this parameter increased from 71 to 84 nm for SeNPs, indicating the interaction between the nano and bio components (dimension of lysozyme is 4.5 × 3 × 3 nm). The polydispersity index (PDI) of the system also changed from 0.029 for the pristine SeNPs (implying narrow size distribution) to 0.30, giving an idea about notable changes in the nanoparticle size distribution upon interaction with lysozyme. Taking the zeta potential value of lysozyme into account (around +6.5 mV), protein adsorption onto Se nanoparticles induced a steady increase in zeta potential of nanoparticles. The zeta potential of untreated SeNPs was estimated to be −30.2 mV, which increased to −3.3 mV due to the adsorption of positively-charged lysozyme molecules onto the matrix of SeNPs (Fig. 2c,d). This represents that electrostatic interactions have played a key role in formation of protein-loaded nanoparticles. It is worth to mention that zeta potential is an important indicator of the stability of colloidal suspensions. Therefore, the negative charge of the protein-loaded nanoparticles implies that the overall system exists in a stable condition^22^.

**Figure 3:**
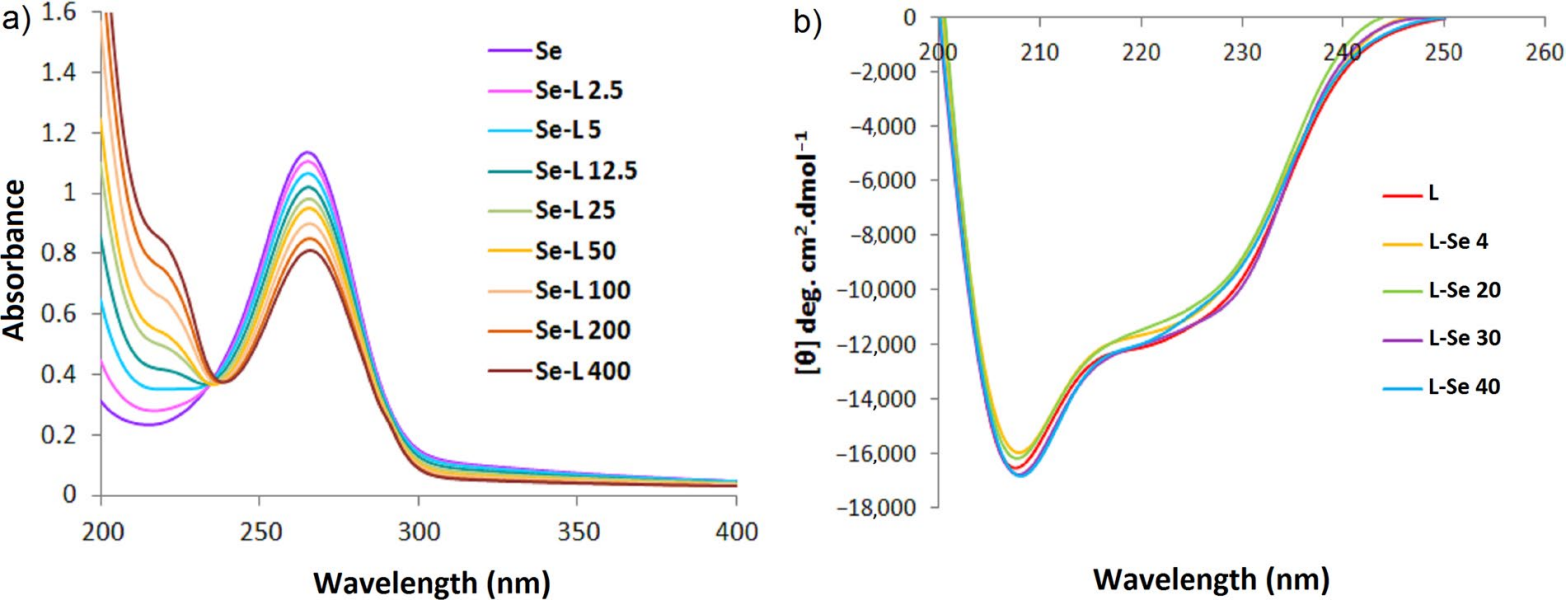
Absorption spectra of SeNPs upon interaction with different concentrations of lysozyme (**a**) and Far-UV CD spectra of lysozyme upon interaction with different concentrations of SeNPs (**b**), in 0.02 mM sodium phosphate buffer, pH 6.2. Se and L represent SeNPs and lysozyme, respectively.

To monitor the interaction of Lysozyme with SeNPs, a fixed concentration of the nanoparticles was incubated with different concentrations of Lysozyme. Absorption spectra of SeNPs are depicted in Fig. 3a. The absorption intensity of the nanoparticles at their characteristic wavelength (265 nm) decreased upon increment of the biomolecule concentration. Nevertheless, the characteristic band has maintained its typical shape, showing that the nanoparticles have not experienced aggregation upon interaction with positively charged biomolecule. Appearance of the samples also confirmed the same (data not shown).

Furthermore, to investigate possible effect of the nanoparticles on the biological component of the Nanohybrid system, Circular dichroism spectropolarimetry of lysozyme was monitored in the presence of different concentrations of the SeNPs. Based on a number of reports, in the context of developing Nanohybrid systems of interest (e.g. for targeted drug delivery purposes), it may be possible that upon interaction and surface adsorption of the biomolecules with nanoparticles some unfavorable conformational changes might occur in the structure of the bio component in the Nanohybrid system, leading to the altered function. On the other hand, there are cases with the nanoparticles in which, structural and functional properties have been improved from biophysical and biochemical aspects. Such facts bring the essence of conducting a series of fundamental studies into notice, before application of any nano-based system for vitro/vivo purposes^20,22,25^.

Figure 3b depicts Far-UV CD spectra and compares the conformation of native lysozyme upon interaction with various concentrations of SeNPs. Circular dichroism is a very helpful technique to monitor induced conformational changes of the macromolecules. A glance at Fig. 3b shows that the characteristic bands of α-helical structure at 208 and 222 nm have negligibly gained less negative value upon interaction with low concentrations of SeNPs (i.e. 4 and 20 µg.mL^−1^), indicating a small decrease in the helical content of lysozyme. Comparison of the CD spectra of lysozyme with higher concentration of SeNPs (40 µg.mL^−1^) with respect to 30 µg.mL^−1^ does not show any notable changes in the secondary structure of the biomolecule. Therefore, as an overall conclusion in this section, lysozyme retained its native structure in the vicinity of SeNPs. This could be a useful data since it could be anticipated that presence of SeNPs as the nano component of the Nanohybrid system may not induce unfavorable effect on the catalytic activity (i.e. antibacterial property) of lysozyme. CD spectrum of the nanohybrid samples after removal of excess protein also shows that the protein bound to the nanoparticles has maintained its native conformation (Fig. S1).

### Antibacterial efficiency of the individual components against *S. aureus*

**Figure 4:**
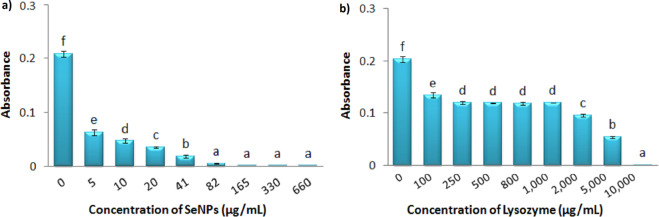
Growth Inhibition of *S. aureus* under treatment with different concentrations of SeNPs (**a**), and Lysozyme (**b**) after 20 hours. Absorbance was recorded at 630 nm. Each bar indicates mean ± standard deviations of three replications. Bars not labeled by the same letter represent statistical significance at P ≤ 0.05 using ANOVA followed by Tukey’s HSD test.

To obtain information about the effective range of inhibition of SeNPs and Lysozyme on the bacterial growth, different concentrations of each component was independently tested against *S. aureus*. Figure 4a shows the growth rate of *S. aureus* under treatment with different concentrations of SeNPs. After 20 hours of incubation, all concentrations were found to effectively reduce bacterial growth. The minimum inhibitory concentration was calculated to be 82 μg.mL^−1^. In order to conduct further experiments, two concentrations of 5 and 10 μg.mL^−1^ of selenium nanoparticles were selected with significant effects (p = 0.000) with respect to the control group.

The same has been tested to monitor the growth rate of *S. aureus* under treatment with different concentrations of Lysozyme (Fig. 4b). Analysis of data showed that within a wide range of concentration (100 μg.mL^−1^ to 1 mg.mL^−1^) of this enzyme there isn’t any notable change in the antibacterial activity of lysozyme. The MIC value in this experiment was estimated to be 10 mg.mL^−1^. In order to investigate the antibacterial efficiency of the Nanohybrid system, the lowest concentration of lysozyme (100 μg.mL^−1^) was selected at a level of significance of p = 0.000 with respect to the control group.

### Antibacterial efficiency of the individual components against *E. coli*

**Figure 5:**
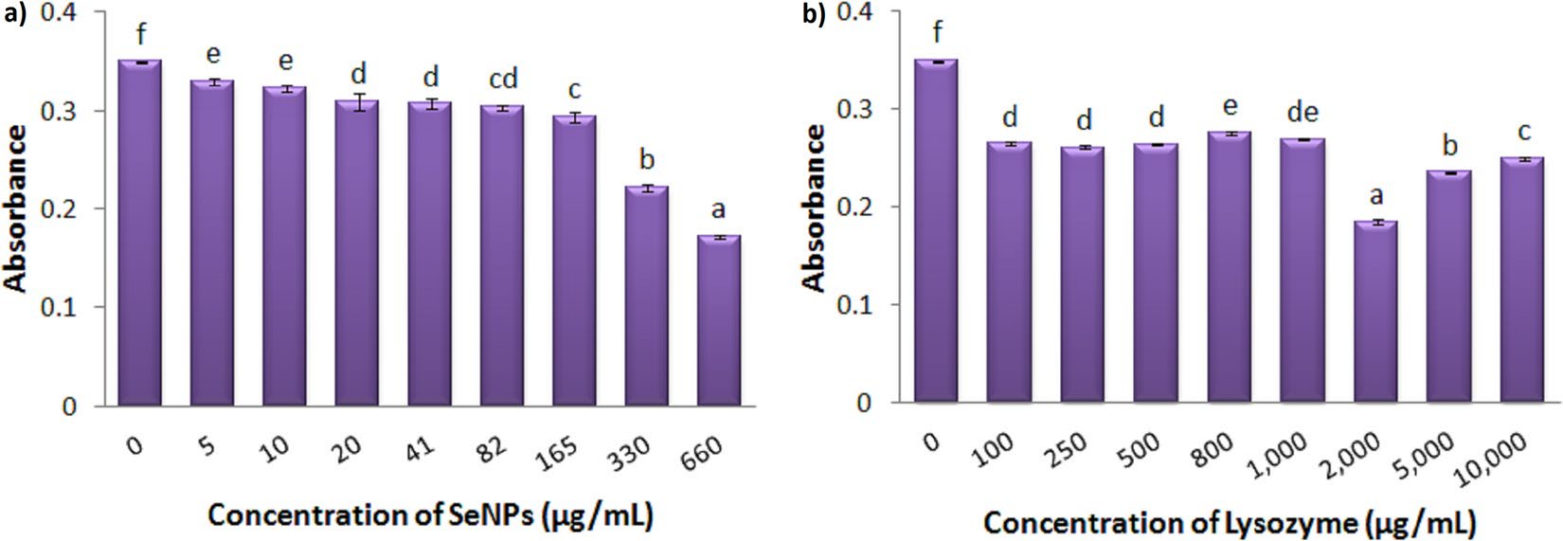
Growth Inhibition of *E*. *coli* under treatment with different concentrations of SeNPs (**a**) and Lysozyme (**b**) after 20 hours. Absorbance was recorded at 630 nm. Each bar indicates mean ± standard deviations of three replications. Bars not labeled by the same letter represent statistical significance at P ≤ 0.05 using ANOVA followed by Tukey’s HSD test.

Figure 5a shows the growth of *E*. *coli* incubate with different concentrations of SeNPs. The gram-negative bacteria showed significant resistance to nanoparticles within a wide range of concentration. The same was observed when lysozyme was tested (Fig. 5b). Within the specified ranges of concentration, neither the nanoparticles nor the protein could completely inhibit the growth of *E. coli* in this study. The highest inhibition was observed at 660 μg.mL^−1^ of SeNPs and 2 mg. mL^−1^ of Lysozyme (41%). To monitor the antibacterial efficiency of the Nanohybrid system, 330 μg.mL^−1^ of SeNPs (at a level of significance of p = 0.000 with respect to the control group) and 100, 2000, 5000 μg.mL^−1^ of Lysozyme (at a level of significance of p = 0.000 with respect to the control group) were selected for further experiments.

### Antibacterial efficiency of the nanohybrid system against *S. aureus and E. coli*

**Figure 6:**
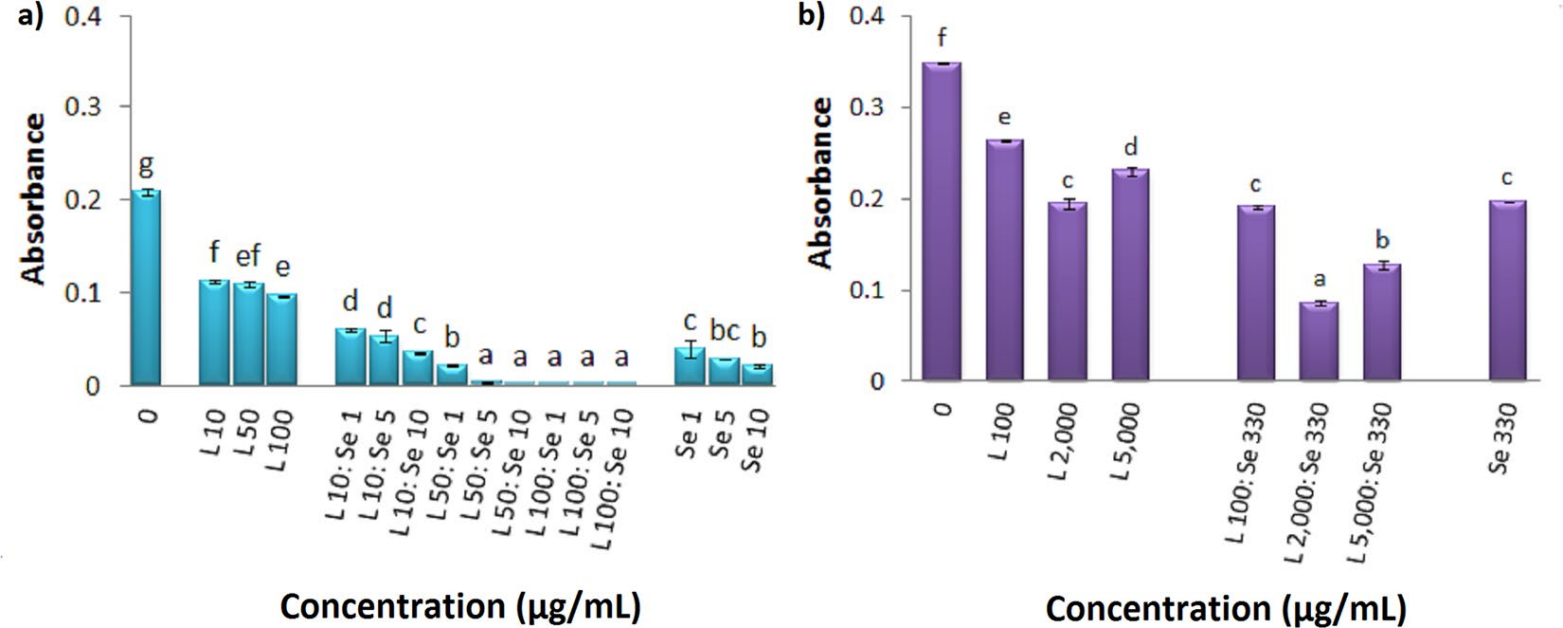
Antibacterial efficiency of Lysozyme, Nanohybrid system and SeNPs against *S. aureus* (**a**), *E*. *coli* (**b**). Absorbance was recorded at 630 nm. Se and L represent SeNPs and lysozyme, respectively. Each bar indicates mean ± standard deviations of three replications. Bars not labeled by the same letter represent statistical significance at P ≤ 0.05 using ANOVA followed by Tukey’s HSD test.

Figure 6a compares the growth of *S. aureus* in the presence of individual components (i.e. SeNPs and Lysozyme) as well as the hybrid nano system. The nanohybrid system with the lowest concentration of lysozyme (10 μg.mL^−1^) has no effect as compared to those of SeNPs controls. Nevertheless, with other low concentrations i.e. 50 and 100 μg.mL^−1^ of lysozyme, the nanohybrid system containing the lowest amount of the nano component have induced complete inhibition of *S. aureus* growth. Upon interaction of all three concentrations of SeNPs (1, 5, 10 μg.mL^−1^) with Lysozyme, the nanohybrid system has efficiently enhanced the antibacterial activity with respect to the protein alone. Therefore, SeNPs play an important role in inhibition of bacterial growth at very low concentration of protein. Another important feature of this hybrid nano system is that lysozyme has also played a vital role in antibacterial property of SeNPs. Although individual Lysozyme (100 μg.mL^−1^) is not remarkably found effective on inhibition of *S. aureus* (see Fig. 4b), its presence in the Nanohybrid system containing 1 μg.mL^−1^ SeNPs has presented ideal synergistic effect and 100% inhibition; whereas the nanoparticle itself has not induced such effect at this concentration.

Hence, it can be concluded that although individual SeNPs have shown to efficiently reduce bacterial growth at low concentrations (MIC of 82 μg.mL^−1^) with respect to very high concentrations of Lysozyme (MIC of 10 mg.mL^−1^), presence of both nano and bio counterparts have induced vital interplay in the Nanohybrid system.

Figure 6b depicts antibacterial property of individual SeNPs and Lysozyme and their hybrid system against *E. coli*. The same trend has been observed in this case and interaction of nanoparticles with the biomolecule has led to improvement in the antibacterial efficiency with respect to the individual components against *E. coli*. The Nanohybrid system with 330 μg.mL^−1^ SeNPs and 2 mg.mL^−1^ Lysozyme is found to have presented the highest level of inhibition (74%).

### Aging and stability study of the nanohybrid system

**Figure 7:**
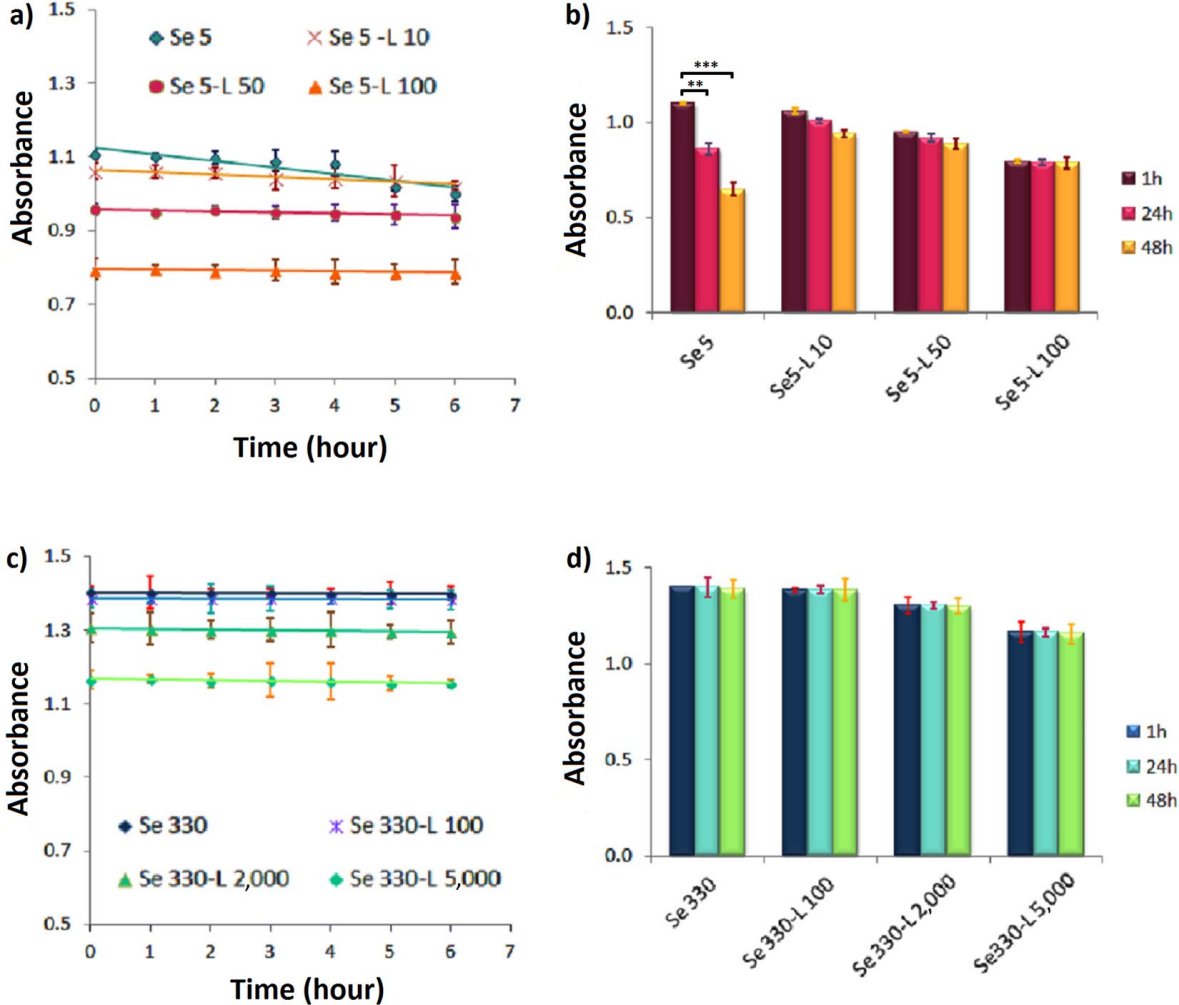
Aging of the pristine and protein-loaded form of SeNPs with two different concentrations of 5 (**a**) and 330 µg/mL (**c**) after 6 h (**a,c**), and 48 h (**b,d**) of incubation with the optimized concentrations of lysozyme. Absorbance was recorded at 265 nm for SeNPs. Se and L represent SeNPs and lysozyme, respectively.

Prior to utilize any nanohybrid systems, it is very important to monitor the stability of the nanoparticles. Many times, upon ageing, the electrostatic interaction itself plays a key role in inducing severe decrease in the interparticle distance, thereby guiding the overall system into irreversible aggregation. Figure 7(a–d) compares stability of selenium nanoparticles as an intact and complex form with lysozyme. Interestingly, at much diluted sample of SeNPs (5 µg.mL^−1^), its complex form with lysozyme seems to provide more stability for the nanoparticles (Fig. 7a). This is better observed when samples are aged up to 48 hours (see Fig. 7b). Upon increment of SeNPs concentration (330 µg.mL^−1^), this is not observed and both the intact and complex form of SeNPs show good stability. It is worth mentioning that the nanohybrid system retains its appearance and the characteristic absorption band even after several weeks of storage under refrigerated condition (Fig. S2).

A glance at the available reports shows that the UV-vis spectrum of SeNPs is in agreement with previous studies^26,27^, and the nanoparticles have been synthesized at a monodisperse state with spherical morphology^7,28^. The integration of different concentrations of nanoparticles with lysozyme did not induce any adverse effect on any of the components and both nanostructures and protein showed good stability and maintained native conformation, respectively. This is the first time that conformation of lysozyme has been monitored upon interaction with SeNPs.

Several studies have shown the antimicrobial activity of selenium nanoparticles which is consistent with our findings^29,30^. The difference in the available results toward growth inhibition may originate from the differences in the synthesis method, size, morphology, purity and concentration of SeNPs, as well as different bacterial strains or methods for evaluation of antibacterial activities.

There is no report to monitor the antibacterial activity of SeNPs in form of a Nanohybrid system with lysozyme to obtain synergistic effects. There are several studies which show improved antibacterial effects when lysozyme is used in combination with other nanomaterials. Ernest *et al*. reported a relative synergistic bactericidal effect between lysozyme and AgNPs compared to antibacterial properties of free lysozyme^31^. In a study conducted by Tripathy *et al*., the MIC value for lysozyme–ZnO nanoparticle conjugate towards *S. aureus* and *E*. *coli* were evaluated to be 18 and 12 μg.mL^−1^, respectively: while for bare lysozyme it was found to be 191 and 236 μg.mL^−1^, respectively^32^. Abu Hamed *et al*., observed the synergistic effect of lysozyme bound to aminated cellulose nanocrystals (AM-CMC) Am-CNC-lysozyme conjugates that inhibited *E*. *coli* growth at 650 μg.mL^−1^ ^33^. Moreover, Wu *et al*., stated that the integration of lysozyme into chitosan nanoparticles (CS-Lys-NPs) increased the antibacterial activity of nanoparticles against E. coli, so that the MIC value of chitosan nanoparticles bound to lysozyme decreased from 5.8 to 5.5 mg.mL^−1^ ^34^. Yu *et al*. also constructed a hybrid coating on titanium implant containing lysozyme (10 mg.mL^−1^), chitosan, silver and hydroxyapatite^35^. Antimicrobial and cell culture tests showed that the Lys/CS/Ag/HAp hybrid coating not only boosted the antibacterial activity of titanium, but also revealed little toxicity to cell activity due to the low Ag content. The antibacterial efficiency of Lys/CS/Ag/HAp-Ti could increase to 95.28% and 98.2% against *E. coli* and *S. aureus*, respectively. Hence, the hybrid coating on Ti implant endowed this material with highly effective antibacterial activity and biocompatibility.

SeNPs with lower cytotoxic effects than those of AgNPs have attracted significant attention these days. Nevertheless, conclusive reports on the role of size, monodispersity, morphology, surface charge, etc. on their antibacterial efficiency are scarce. Moreover, there is not enough record to monitor and compare the possible synergistic effect of these nanoparticles in hybrid systems. A very recent study conducted by Jamroz *et al*., reported incorporation of SeNPs and AgNPs into furcellaran-gelatin (FUR/GEL) films^36^. They observed that FUR/GEL films containing SeNPs showed greater antimicrobial activity against Escherichia coli, Staphylococcus aureus and MRSA than films containing AgNPs. Therefore, it can be assumed that SeNPs can be a promising candidate for the replacement of AgNPs in the upcoming antibacterial applications.

Results of the antibacterial tests on *S. aureus* and *E*. *coli* indicated that nanoparticles, lysozyme and also the hybrid system showed great inhibitory effect on the growth of Gram + bacteria as well as the growth of Gram- bacteria (at higher doses). Since lysozyme acts as a β-glucosidase enzyme to hydrolyze 1, 4-beta-linkages in the cell wall of some microorganisms, it can be concluded that lysozyme is an enzyme effective on gram + bacteria. Meanwhile, the lipopolysaccharide (LPS) layer in Gram- bacteria protects them against this protein^37,38^.

The mechanism of the cytotoxicity of SeNPs against bacteria has still remained unclear. Zeta potential of nanoparticles is important in interacting with bacteria. Many bacteria have a negative surface charge. Due to the additional layer of negatively charged lipopolysaccharides, Gram negative bacteria are more negatively charged than Gram positive bacteria^39,40^. Therefore, it is expected that strong repulsive forces exist between SeNPs and highly negative charged bacteria such as *E*. *coli*. For bacteria with a lower (or neutral) surface net charge, such as *S. aureus*, the interaction between SeNPs and bacteria can help nanoparticles to deposit on the membrane of the bacteria, which can have adverse effects on the cell division or bacterial survival^7^. Overall, difference in the cell wall composition and the surface characteristics of two types of bacteria contributes to the difference in the inhibitory effects between gram- and gram + bacteria. In this research, the negative charge of the hybrid system is reduced compared with nanoparticles and consequently this results in less electrostatic repellency when exposed to bacteria. This is probably one of the reasons that acknowledge the synergistic effect of the hybrid system.

## Conclusion

This effort investigated the antibacterial activity of selenium nanoparticles in form of a nanohybrid system. SeNPs were incubated with Lysozyme and the antimicrobial property of the nanohybrid system was monitored against *Escherichia coli* and *Staphylococcus aureus*. The MIC value for SeNPs against *Staphylococcus aureus* was found to be 82 μg.mL^−1^; whereas very high concentrations of Lysozyme were required for 100% inhibition (10 mg.mL^−1^). Upon interaction of SeNPs with the biomolecule, both *nano* and *bio* counterparts induced vital interplay in the nanohybrid system with very low concentrations, leading to synergistic effect on the antibacterial property against both bacterial strains. Study of the aged samples of the nanohybrid system also revealed good stability of SeNPs both as the intact and complex form. Results of this effort encourage utilization of nanohybrid systems with synergistic antibacterial properties for a variety of applications in medicine, biomedicine, food safety and health care products. Nonetheless, more investigations are required to fully elucidate the mechanism and effects of cytotoxicity and antibacterial properties of such types of nanohybrid systems.

## Methods

### Chemicals and bacterial strains

Lysozyme from chicken egg white was purchased from Bio Basic. Sodium selenite, Sodium dihydrogen phosphate dehydrate, Disodium monohydrogen phosphate were procured from Merck. Ascorbic acid and Polysorbate 20 were purchased from Sigma. Muller-Hinton broth/agar were purchased from HiMedia. *Escherichia coli* (ATCC25922) and *Staphylococcus aureus* (ATCC25923), were obtained from the department of Bacteriology of Tarbiat Modares University, Iran. Ultrapure deionized water (DI) was used during the whole experiments. Glassware was thoroughly cleaned with dilute sulfo chromic acid/detergent solution and rinsed with DI.

### Synthesis of selenium nanoparticles

SeNPs were prepared via a reduction of sodium selenite by ascorbic acid and stabilized by polysorbate 20. Briefly, 30 mg of Na_2_SeO_3_.5H_2_O was added to 90 mL of Milli-Q water. Ascorbic acid (10 mL, 56.7 mM) was added dropwise to sodium selenite solution with vigorous stirring.10 µL of polysorbate were added after each 2 ml of ascorbic acid. Selenium nanoparticles were formed after the addition of ascorbic acid. This can be visualized by a color change of the reactant solution from clear white to clear red. All solutions were made in a sterile environment by using a sterile cabinet and double distilled water. Selenium nanoparticles were then collected by centrifuging the solution at 12000 rpm. The pellet was resuspended in sterile double distilled water before use in bacteria experiments. Selenium contents of nanoparticles were determined using inductively coupled plasma optical emission spectroscopy (ICP-OES, model Vista-Pro from Varian).

### SEM and UV–Vis characterization

Size, morphology and distribution of SeNPs were studied by scanning electron microscopy (SEM). Dilute sample was prepared and well dispersed by sonication. Samples were then dried and labelled with a gold monolayer (sputter coating), imaged by Qunta 200 electron microscope. SeNPs were also characterized by UV–Vis absorption spectrophotometer (PerkinElmer, Lambda 25). The spectra were recorded within 200–500 nm wavelength range.

### Nanoparticle-protein interaction

SeNPs were mixed with different concentrations of protein in sodium phosphate buffer (20 mM, pH 6.2). Samples were then incubated at ambient temperature for 1 h prior to characterization. For analysis of protein conformation, a fixed concentration of lysozyme (200 μg.mL^−1^ in phosphate buffer 20 mM, pH 6.2) was mixed with various concentrations of SeNPs at ambient temperature. Longer incubation time did not affect the interaction. All experiments were performed with the as-prepared nanohybrid system.

### Dynamic light scattering

Effects of protein concentration on the average hydrodynamic size of nanoparticles were monitored using dynamic light scattering. The solvent should be of high purity (MiliQ water), without large molar mass impurities (e.g. dust) to avoid “shadowing” of the signal for the scattering particles. Surface charge of the nanoparticles was determined using Malvern Zetasizer Nano-ZS instrument.

### Circular dichroism spectropolarimetry

Circular dichroism (CD) measurements were carried out on a JASCO spectropolarimeter (J-715) to monitor possible changes that SeNPs might induce in lysozyme’s conformation. Far UV-CD region was scanned in the wavelength range of 200–250 nm. CD is reported in terms of mean residue ellipticity [θ] in deg.cm^2^.dmol^−1^, as depicted in the following equation:

1 $$[{\theta }]=100\,[{\theta }]\,{\rm{Mw}}/{\rm{ncl}}$$

Where, θ is molar ellipticity, Mw is the lysozyme molecular weight, n is the number of amino acid residues, c is the lysozyme concentration in mg.mL^−1^, and l is the light path length in centimeters. Data was smoothed and analysed by Jasco software, after subtracting the buffer and SeNPs contribution from the original protein and protein–SeNPs complex, respectively.

### Antibacterial activity test of nanohybrid system

SeNPs, Lysozyme and nanohybrid system were tested for minimum inhibitory concentration (MIC) leading to the inhibition of bacterial growth by standard microdilution method (provided by the CLSI, 2018). MIC is the lowest concentration of an antimicrobial agent that prevents visible growth of a microorganism in an agar or broth dilution susceptibility test. A 96-well microtiter plate dilution was applied to determine the MIC. Briefly, 50 µL of twice the desired final concentration of antimicrobial agent was added to each well containing 50 µL Muller-Hinton broth. The inoculums were prepared by using direct colony suspension method by making a direct saline suspension of isolated colonies selected from a 24 h agar plate and then adjust the suspension to achieve a turbidity equivalent to a 0.5 McFarland standard. A total of 10 µL of activated culture of each tested strain (about 5 × 10^6^ CFU/mL) was added. After 20 h of incubation at 37 °C, bacterial growth inhibition was determined by monitoring the optical density at 630 nm by ELX 808 microplate reader (Biotek Instruments, Winooski VT, USA). Absorbance at 630 nm showed the bacterial growth. The MIC value was recorded as the lowest concentration of the sample inhibiting the visible growth of microorganisms. A blank control without the sample was used for the control

### Statistical analysis

All quantitative tests were carried out over three times and then mean values with standard deviations were calculated. Statistical analyses were performed using SPSS 16 software. Data were collected, and the significant differences were assessed with the probability associated with one-way ANOVA tests. One-way ANOVA followed by Tukey’s multiple-comparison post hoc tests were exploited for the comparison of three or more groups. The significance level (*p*) was considered at 0.05.

## Supplementary information

Supplementary Figures S1 and S2.

## Data Availability

All raw and analyzed data as well as the materials are available in this study.
