# Supplementary material for: Synthesis and Characterization of Selenium Nanoparticles-Lysozyme Nanohybrid System with Synergistic Antibacterial Properties
Source: Sci Rep. 2020 Jan 16;10:510. doi: 10.1038/s41598-019-57333-7 (PMC6965607; doi:10.1038/s41598-019-57333-7)
Supplement: Supplementary file 1 — Supplementary Figures S1 and S2. [file 41598_2019_57333_MOESM1_ESM.pdf]

Supplementary Figures

Synthesis and Characterization of Selenium Nanoparticles-Lysozyme Nanohybrid System with Synergistic Antibacterial Properties

Mahsa Vahdati[1], Tahereh Tohidi Moghadam[2*]

1. Department of Biology, Science and Research Branch, Islamic Azad University

2. Department of Nanobiotechnology, Faculty of Biological Sciences, Tarbiat Modares University

*Corresponding Author: t.tohidi@modares.ac.ir

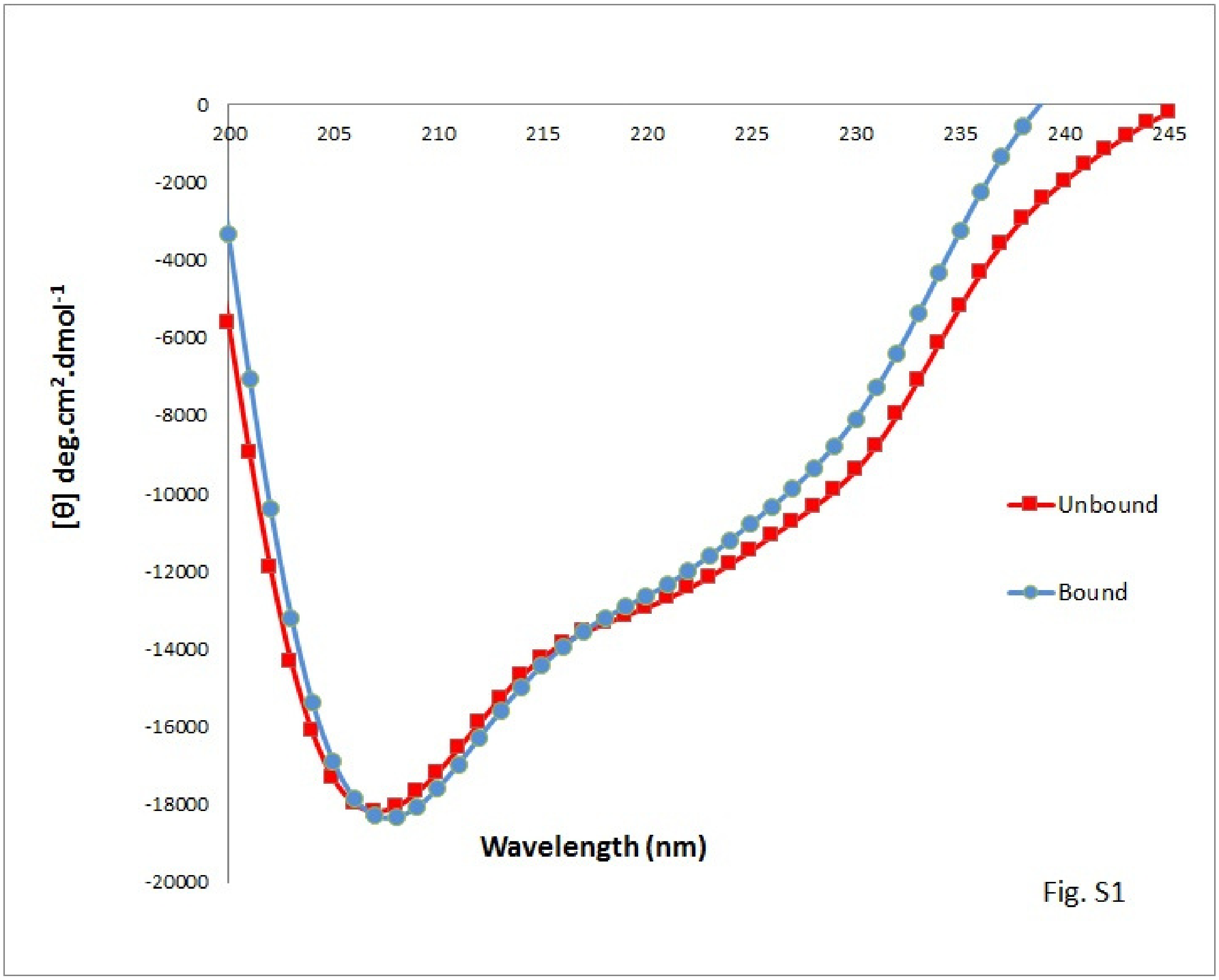

Fig. S1. Comparison of Circular dichroism spectropolarimetry of lysozyme in untreated and bound from (present in the purified nanohybrid samples). Final concentration of SeNPs in the hybrid system is 40 µg. mL^{-1}).

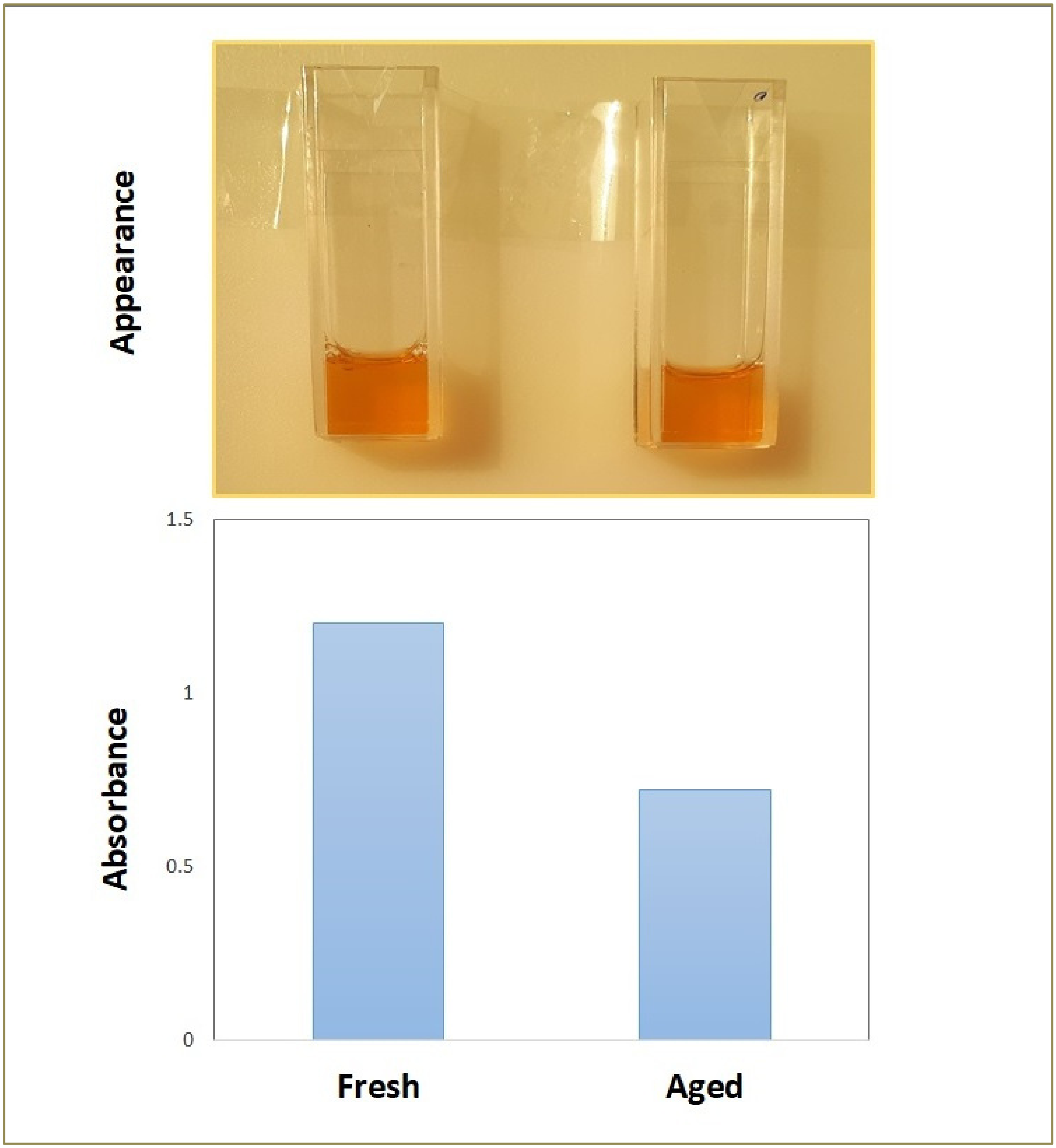

Fig. S2. Appearance and absorbance of the aged nanohybrid system at 265 nm, after six weeks of storage under refrigerated condition.
